# A Concise Review on Epigenetic Regulation: Insight into Molecular Mechanisms

**DOI:** 10.3390/ijms12128661

**Published:** 2011-11-30

**Authors:** Shahram Golbabapour, Mahmood Ameen Abdulla, Maryam Hajrezaei

**Affiliations:** Department of Molecular Medicine, Faculty of Medicine, University of Malaya, Kuala Lumpur 50603, Malaysia; E-Mails: ammeen@um.edu.my (M.A.A.); maryam_hajrezaie@yahoo.com (M.H.)

**Keywords:** epigenetic, pluripotency, SCNT, embryogenesis, gametogenesis, polycomb, methylation, histone modification

## Abstract

Epigenetic mechanisms are responsible for the regulation of transcription of imprinted genes and those that induce a totipotent state. Starting just after fertilization, DNA methylation pattern undergoes establishment, reestablishment and maintenance. These modifications are important for normal embryo and placental developments. Throughout life and passing to the next generation, epigenetic events establish, maintain, erase and reestablish. In the context of differentiated cell reprogramming, demethylation and activation of genes whose expressions contribute to the pluripotent state is the crux of the matter. In this review, firstly, regulatory epigenetic mechanisms related to somatic cell nuclear transfer (SCNT) reprogramming are discussed, followed by embryonic development, and placental epigenetic issues.

## 1. Introduction

**Figure 1:**
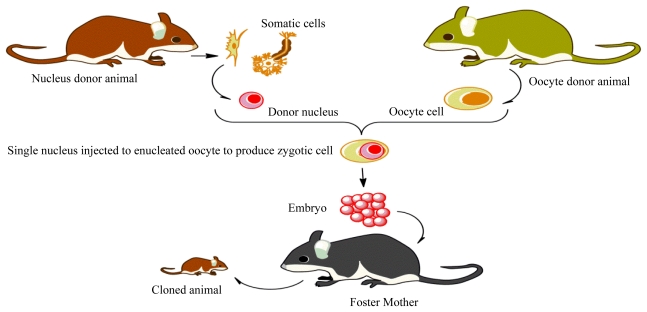
The schematic method used to create a cloned animal. A nucleus is taken from a somatic cell (nucleus donor animal) and injected into enucleated Oocyte (Oocyte donor animal). The zygotic cell begins dividing and the resultant blastocyst (embryo) transfers to a foster mother to develop the cloned animal.

The transition from the differentiated somatic cell to the embryonic stage through somatic cell nuclear transfer (SCNT) requires activation energy to efficiently reprogram the resultant zygote to a proper pluripotent state [1,2]. SCNT is a tool to clone nuclear material into the enucleated cytoplasm of an unfertilized oocyte and thereby create genetically identical animals (Figure 1). SCNT not only benefits agricultural applications, but has the potential for great advances in the field of medicine. In addition, SCNT has paved the way to better understand the changes in cell differentiation and reprogramming. Despite many investigations that have been done by numerous laboratories, the efficiency (*i.e.*, the ability to create a live born animal per nuclear transfer) by this technique is still below 5% and several abnormalities have been reported [3]. One of the main reasons for these abnormalities is the failure in reprogramming/remodeling of differentiated cells to the stage that will evolve to a normal neonate. In the other words, programs involved in differentiated cells should be replaced with totipotency to ensure nuclear cloning and production of healthy offspring. Gene regulatory pathways are the critical network that could redefine SCNT. Clones, on the other hand, have to change expression profiles to embryo-specific, global rearrangement of chromatin structure. As a result, the cloning study is a way to understand epigenetic mechanisms and reprogram differentiated nuclei. Epigenetic modifications in the donor cells remodel the gene expression profile to the extent that is similar to the normal embryo. However, the epigenetic mechanisms that are responsible for the transformation from a differentiated somatic cell into a pluripotent state remain mysterious. In this review, we explore the epigenetic regulatory events that occur during the gametogenesis, embryogenesis and placental development. The epigenetic modifications that modulate expression of genes and subsequent reprogramming of the somatic nucleus to pluripotent state are also briefly discussed. The purpose of review is to summarize effective epigenetic events that could increase efficiency of SCNT and to emphasize recent epigenetic findings. In this regards, we briefly look into transition techniques and highlight epigenetic modifications that happen during the nucleus reprogramming.

## 2. Transition to Pluripotency

SCNT provides new insight into gene manipulation to achieve defined purposes. This technique is to reprogram the differentiated somatic cell to a pluripotent state by transferring the nucleus of a somatic cell into an enucleated oocyte and produce a zygote, which results in a live offspring. In mammals, genomes of differentiated cell have to reprogram to a totipotent state to establish SCNT during pre-implantation. Consequently, the development of a zygote initiates and follows with blastocyst and the subsequent embryonic stages. Cloned embryos derived from less differentiated cells (as nucleus donors), such as embryonic stem cells, show better implantation than those derived from more differentiated somatic cells probably due to minimum or no reprogramming requirement [4]. It was shown that the efficiency of bovine SCNT is relatively higher than the other experienced species (for see review [5]) and pregnancy in *Bos taurus* is very similar to that of human in terms of length and development.

Generation of induced pluripotent stem cell is to transport defined regulatory signals, influence the epigenetic state and change it to another state (plasticity) which emphasizes the mutual reliance between cell identity and epigenetic states [6,7]. This is, especially true during early embryo development and gametogenesis [1]. Pluripotent stem cells are driven from somatic cells that are introduced by specific reprogramming factors through either cell fusion or delivery of defined biochemical and/or chemical factors which are also categorized as a reprogramming approach. The fusion technique produces hybrid cells from differentiated somatic cells by nuclear reprogramming through the reactivation of embryo-specific genes, whose expressions are suppressed in somatic cells [8]. In 2006, a four-gene set was introduced to reprogram somatic cells to a pluripotent state [7]. Hybrid cells produced by fusion technique show a pluripotent state by expression of the pluripotent markers such as *OCT4* [9]. Moreover, a number of other genes such as *Nanong*, *Sox2*, *Lin28*, *Klf4*, *c-Myc* and *AID* have been correlated to the pluripotent state of a cell. The expressions of these genes result in cell reprogramming [7,10–13]. Based on these evidences, identification of embryo-specific genes is crucial to defining their expression profiles during embryogenesis, functions during different stages of embryogenesis and the development of placenta. These regulations, actually, are defined epigenetic regulation for which molecular signals modulate the modifications.

Several morphological abnormalities such as hydroallantois, placentomegaly, cardiomegaly, enlarged umbilical cord, abdominal ascites and placental dysfunctions [14,15], have been observed in the cloned offsprings. Large offspring syndrome (LOS) is a developmental disorder mostly seen in SCNT driven embryos. This syndrome in addition to the failure in the development of embryo and placenta and other abnormalities is attributed to inappropriate and/or inadequate somatic nuclear reprogramming events. Significant increase in genomic methylation in liver of cloned bovine fetuses is attributed to fetal overgrowth [16]. LOS and failure in the normal development of an embryo that are seen in cloned animals could be due to abnormal epigenetic patterns [17]. In fact, assisted reproductive techniques appear to be accompanied by several anomalies, especially in the second half of the gestation [14,18–20].

## 3. Molecular Signals in Epigenetic Regulation

Cells’ information is inherited to the next generation through genetic and epigenetic routes. Genetic information is encoded in the DNA sequence while, epigenetic information is defined basically by DNA modification (DNA methylation) and chromatin modifications (methylation, phosphorylation, acetylation and ubiquitination of histone cores). Combination of these modifications characterizes the chromatin configuration and the accessibility of genes to the transcription machinery and consequently, transcriptional regulation of the expression of genes. Cheng [21] introduced three categories by which transcriptional function is generally initiated and controlled: First, general intrinsic promoter and transcriptional machinery [22–24], second, specific transcriptional regulatory factors [25–27] and, third, the configuration and accessibility of chromatin structure and DNA to the transcriptional machinery through posttranslational modifications of histone and post replicational modification of DNA [27–29].

### 3.1. Main Epigenetic Regulatory Mechanisms

Complex epigenetic regulation comprises several molecular signals that direct the expression of genes based on environmental changes and developmental status. Transcription factors, non-coding RNAs (ncRNAs) [30], DNA methylation, histone modification and chromatin remodeling are such epigenetic signals that mediate accessibility and expression of genes as needed. Transcription mainly defines a self-propagating state mediated by *cis*-acting and/or *trans*-acting regulatory mechanisms [31], and are able to establish epigenetic states through *cis*-acting [32] and non-coding polycomb domains [31,33]. Reinforcement of epigenetic states happens through mutual relationship between DNA methylation and histone modifications [34]. DNA methylation postulates a reinforcing signal for other regulatory mechanisms whose functions are not that much strong [31].

DNA methylation and histone modification are two important mechanisms for modulating the chromatin structure and regulating the expressions of the genes (for review see [35]). Epigenetic regulation is a complex phenomenon that consists of a variety of different processes [21] such as imprinting [36], X chromosome inactivation [37] and gene silencing [38,39]. In addition it encompasses the development of an embryo [40–43] and placenta [44–46], nuclear reprogramming in SCNT embryos [3,6] and carcinogenesis [47,48].

### 3.2. Transcriptional Regulation

The transcriptional regulation of genes is mainly directed by different strategies. These include the state of genomic methylation [21], chromatin configuration [49,50], chromatin structural variations (euchromatin and heterochromatin) [51,52], and chromatin modifications [53]. Chromatin modification in turn is influenced by methylation, acetylation and phosphorylation, as well as polycomb proteins [54] and matrix attached region [55]. Transcriptional regulation is mostly controlled by the methylation pattern of the genome. DNA methylation on specific CpG dinucleotide (CpG) located in a cluster (CpG islands) is the regulatory mechanism by which expression of gene is either activated or suppressed (for review see [56]). Moreover, chemical modifications of chromatin histone cores are mediated by DNA methylation of CpG islands [57]. These modifications have a mutual relationship with each other [58]. Germ cells and embryonic cells during early development are two epigenetic sites where methylation patterns erase, establish and reestablish [59].

### 3.3. Epigenetic Reprogramming During Embryogenesis

In mammals, epigenetic reprogramming in germ cells and during preimplantation, especially its effects on imprinting genes, predominantly establishes developmental stages [60]. The DNA methylation patterns characterize developmental status during cell differentiation. In the concept of epigenomics, molecular signals are responsible to establish the proper expression of embryo-specific genes, mainly during gametogenesis and embryogenesis. Therefore, the main issue for a successful SCNT is the establishment of these modifications, occurring during embryogenesis, which should be similar and ideally identical to its normal embryo counterpart. However, undoubtedly, several lessons are still to be learnt regarding epigenetic modifications during gametogenesis.

### 3.4. Epigenetic Features of DNA Methylation

As mentioned before, DNA methylation and histone modification are the main epigenetic factors, by which gene expression could be regulated, and have important roles in nuclear reprogramming during embryogenesis. DNA methylation is a heritable epigenetic marker by which expression of a gene may be regulated through alteration in the local chromatin structure that mostly happen within the CpG islands and imprinted genes at cytosine carbon 5 within palindromic dinucleotide 5′-CpG-3′ and differentially methylated domains (DMDs) respectively (see review [60]). Cytosine residue of CpG is the site for DNA methylation by which gene expression is regulated. Generally, DNA methylation at CpG sequences suppresses the expression of the methylated gene [61]. CpG islands are usually located within repetitive elements such as centromic repeats, satellite sequences and ribosomal RNA genes [62,63]. DNA methylation can be varied in terms of patterns and the level of global/regional DNA methylation, is specific to developmental stages [64] and origin of tissue [16,65]. In fact, the mature parental gametes at fertilization are significantly methylated. For instance, DNA of sperm, in comparison with that of the oocyte, is more methylated [66,67] and, undergoes demethylation after fertilization [68–70]. However, imprinted genes and some retrotransposons mostly remain methylated. In mouse, hypermethylation pattern in repetitive regions and heterochromatin region has been observed; whilst in gene-specific region of DNA hypomethylation is predominant [17,71,72]. Abnormal DNA methylation of various repetitive elements in cloned blastocysts was reported for the first time by Kang and coworkers [73]. Methylation of imprints, monoallelic expressed genes [74,75], on the other hand, is a maintained (not *de novo*) and highly conserved event [76,77]. Recently, in a human study, comparison between embryonic stem cells and differentiated cells illustrated that there are a number of methylated cytosine in non-CpG regions of the embryonic stem cells [78].

### 3.5. DNA Methylation Signals

DNA methylation is under the control of two types of signals: *cis*-acting signals and *trans*-acting signals. *IGF2R*, *SNRPN, H19* and *RASGRF1* are genes regulated by *cis*-acting signals (see review [79]). Global DNA methylation takes place especially after fertilization and with different rate of demethylation that is specific to either parental genome [61]. Cell cycle observations reveal that paternal demethylation generally happens during the first cell cycle but maternal alleles take a few cycles to be demethylated [80]. After fertilization, imprinting control regions (ICRs) methylation is established in a sex-dependent manner [61]. Despite the maintained methylation pattern in somatic cells, methylation pattern in germ cells needs to be appropriately reestablished to provide a methylation pattern that is heritable to the next generation. This suggests that methylation is modulated in a sex-dependent manner [81]. Although hypomethylation of female germ line seems not to be correlated to the sex chromosomes, their regulation is thought to be associated with genital ridge. However, in the male germ line it is regulated by both mechanisms [81,82].

### 3.6. DNA Methylation Analysis

Epigenetic studies are strongly involved in DNA methylation. Analysis of the methylation patterns is the main approach in different studies that focuses on gene regulation. The cytosine 5 methylation in the context of CpGs mostly takes place within CpGs islands at the promoter region of genes and this leads to suppression of the expression of the gene. Several studies correlate aberrant methylation pattern of DNA to developmental failure during embryogenesis [83–85] and placentation [86,87] as well as several diseases and disorders [88–91] (for review see [92]). DNA methylation techniques cover a wide range of analysis from gene specific, locus specific to entire genome analysis using proper methods categorized in four groups based on DNA methylation analysis techniques [93]: in the first category cytosine residues are converted to Uralic by a bisulfite conversion, the second category is based on methylation-sensitive restriction endonuclease, enrichment based methods and the last is the capturing method based on the affinity to retain methylated DNA [94,95].

### 3.7. Regulatory Factors in DNA Methylation

As mentioned before, DNA methylation is a common epigenetic modification taking place by enzymatic reactions to be added to the cytosines at CpG, mostly known as repetitive elements and imprinted genes [79]. Generally, DNA methylation is classified either as *de novo* methylation or maintained methylation. Therefore, there are two classes of enzymatic: *de novo* methyltransferases and maintenance methyltransferases [96,97]. In mammals, DNA methylation occurs by the addition of a methyl group from S-adenosylmethionine to Cytosine using DNA methyltransferases (DNMTs). DNMTs are *trans*-acting factors targeting DNA sites for methylation using *cis*-acting signals. There are a number of mammalian DNMTs (see Table 1) that have been identified since 1980s [98] (for review see [99]).

### 3.8. DNA Methyltransferases

DNA methylation at cytosine 5 nucleotide is catalyzed by DNMTs. This family of enzyme is vitally important in epigenetic regulation which modulates the expression of genes especially imprinted ones as well as X chromosome inactivation [114,115]. There are five main DNMTs that are important in *de novo* and/or maintenance DNA methylation: DNMT1, DNMT10, DNMT3a, DNMT3b and DNMT3L [99]. DNMT1, DNMT2 and DNMT3 are mostly characterized DNMTs that can categorize either maintenance or *de novo* DNMT. DNMT1 is a maintenance DNMT that methylates both imprints and non-imprints genes. DNMT2 seems to have a regulatory role in DNA methylation but the mechanism and its role in methylation maintenance or *de novo* remains unclear. DNMT3 as a *de novo* DNMT (DNMT3a) is a key factor in imprints’ methylation. Its isoforms are suggested to have roles in global DNA methylation in germ cells [116].

DNMT3L is defined as an imprints’ regulatory candidate for DNA methylation by regulating NMT3a/b [117]. The expression of *DNMT1* gene has a positive correlation with DNA methylation status on the satellite I region. Consequently, it has been shown that *in vitro* development of bovine SCNT embryos to the blastocysts state can be enhanced through down regulation of DNMT1 [118]. It is also shown that the DNMT is responsible for ICRs methylation [109]. Moreover, transcriptional analysis on the pS2/TFF1 during cell cycles reveals that DNMTs carry out two distinct actions, namely methylation and demethylation of CpGs [119].

In comparison to the male germ line in which the establishment of ICR methylation of an imprinted gene, *H19*, is regulated by DNMT3a and DNMTL [109,113], DNMT3L is the *de novo* methylating regulatory factor for the female germ line [109]. In the female germ line, DNMT3L establishes the methylation of ICRs that selectively interact with histone H3 [120]. This evidence in addition to DNMT3L’s stimulating role for DNMT3a and DNMT3b [117] shows its potential in chromatin mark-specific recognition and methylation establishment [61]. Promoter methylation-mediated DNMTs show down regulation of DNMT1 and up regulation of DNMT3L in the human placenta and brings strength to the capability of DNMT family in the establishment of *de novo* DNA methylation in extraembryonic tissue [46]. A novel DNMT3b splice variant, DNMT3B3Δ5, is highly expressed in pluripotent embryonic stem cell and in contrast, is repressed during differentiation [121].

In a human study on DNMTS, global hypomethylation is shown to be induced by significant reduction in the expression of *DNMT3A*, *DNMT3b* and *DNMT1* using microRNA-29b [122]. DNMT3L by itself has no methyltransferase activity; however, its association with the DNMT3 family seems essential for *de novo* methylation in mice [123]. There are some evidence on the activity of DNMT1 in the establishment of methylation at non-CpG regions [55] and CpG islands [21,124,125]. Histone modification and CpG spacing are able to direct DMR methylation of imprints [21]. Crystallographic analysis showed that *de novo* DNA methylation might be controlled by specific histone modifications in that a heterotetramer structure, assembled from DNMT3a and DNMTL, provides two active sites of CpG that are 8–19 base-pair distance from each other [126–128]. A study on chromosome 21 also Reinforces the crucial role of CpG spacing in DNA methylation [129]. Active demethylation in mammalian genome seems promising, however, there has been no report of an enzyme that can catalyze this reaction [130]. Some studies have emphasized an active demethylation process, independent from DNA replication [131,132]. In fact, demethylation of the paternal alleles is an active event that happens rapidly after fertilization. The maternal genome, however, demethylates during first cell cycles in which demethylation mostly appears to be an inactive process. In addition to DNA methylation and histone modification, ncRNAs and regulatory proteins are the most studied epigenetic mechanisms that modulate epigenetic reprogramming. Small interfering RNAs (siRNAs) transfection is a technique to silence DNMT mRNA and modify the DNA methylation pattern in cells [6]. In a recent study, To examine the efficacy of the technique in SCNT embryos, DNMT1 RNA was silenced using siRNA in SCNT bovine embryo which demonstrated the capability in nucleus reprogramming through inducing DNA methylation [118]. In the expression of *H19* in the male germ line, DNMT3a and DNMTL are counterparts and reached their maximum [133,134] on embryonic day 13 while there is no methylation on the *H19* ICR, in mouse [135]. In addition, these enzymes seem not to be specific for DNA binding [99], suggesting direct/indirect interactions with specific chromatin modifications [61,136,137].

### 3.9. Epigenetic Features of ncRNAs

The cluster-oriented imprinted genes are laid in ~1Mb length base pair, containing parental expressed genes, ncRNA sequences that regulate the nearby imprinted genes [138–141]. ncRNAs are mostly placed in clusters and regulated by ICRs [142]. The *GNAS* and *KCNQ1* are examples of such imprints; containing ncRNAs that mediate the gene expression [143,144]. ncRNAs are divided into two groups, small ncRNAs and long ncRNAs. Small ncRNAs attach chromatin modifiers to specific genome sequence [145] and may interact with either RNA, single stranded DNA or double stranded DNA [1,146]. Long ncRNAs have complex tertiary structure and act globally to bridge chromatin modifiers to the genome [147]. But there are some evidences for local function of long ncRNAs, which is considered to function in *cis*-acting regulation of parental imprinted gene and inactivation of chromosome X [1].

In mammalian transcription of ncRNA genes is an important feature. ncRNAs usually are classified based on their mature length, location and orientation according to the nearest protein-coding gene, and their function which could be *cis* or *trans* [148–150]. Macro RNAs, such as inactive X-specific transcript (Xist) and X (inactive)-specific transcript, antisense (Tsix), are categorized as *cis-*acting ncRNAs that usually locate within clusters of imprinted genes. On the other hand, short ncRNAs such as short interfering RNAs, micro RNAs, piwi-interacting RNAs and short nucleolar RNAs are categorized as *trans*-acting ncRNAs (for review see [151]) [148]. Koerner (2009) concluded that chromosomes express macro ncRNA usually do not express imprinted mRNA genes and the expression of imprinted macro ncRNAs may be regulated by an unmethylated imprint control element [148]. Moreover, there are a number of evidences that show *trans*-acting regulators for imprinted small ncRNAs such as *Snurf*-*SNRPN* and *Dlk1*-*Gtl2* [152,153]. It has been shown that ncRNAs have a critical role during development. For instance, two ncRNAs, *Dicer1* and *Dgcr8*, show developmental impact in mice [154,155]. Moreover, studies on effects of ncRNAs during animal embryogenesis show their specific and crucial role during embryonic development (for review see [156]). Micro ncRNAs, specifically, show precise control on expression of imprinted genes during development [157]. For instances, *miR-15* and *miR-16* are important in early embryonic development [158], *miR-1*, *miR-133* and *miR-206* in development of skeletal and heart muscle [159,160], *miR-124* in neuronal development [161] (for review see [162]). During placentation, ncRNAs such as *KCNQ1OT1*, a long ncRNA, also illustrate a leading role in imprinted genes regulation [163–165]. Regulation of ncRNAs is an important silencing mechanism in plancenta (for review see [148]). It was shown that the repression of imprinted genes during gestation is directly regulated by micro RNAs during placentation and embryogenesis [166,167]. It seems that ncRNAs targets placental histone methyltransferases by ncRNAs through chromatin modification [148].

### 3.10. Epigenetic Features of Small RNAs

Small RNAs (terminologically different from ncRNAs), generated by activity of RNaseIII enzymes (reviewed in [168]), have variety of biological functions such as heterochromatin formation, mRNA inactivation and transcriptional regulation [169,170]. Generally, their bioactivity is due to their association with Argonaute (Ago)-family proteins [171]. microRNAs (miRNAs), endogenous small interfering RNAs and Piwi-interacting RNAs (piRNAs) are classes of small RNAs. In mammalians, small RNA-associated Ago proteins are mostly classified into Piwi subfamily and Ago subfamily (for review see [171]). miRNAs to do their biological activity, which is post translational regulation by acting on mRNAs, needs to be bound by Ago subfamily proteins (for review see [170]). Moreover, regulation of most miRNAs may control by developmental signaling [172]. piRNAs are mostly bound by Piwi subfamily proteins and have a critical role during gametogenesis [173] in germ line [174]. This subfamily protein has shown to have a critical role in regulation of germline stem cells [175].

### 3.11. Epigenetic Features of Chromatin Modifications

Chromatin structure is crucial for gene regulation/expression, which is carried out by exploiting recruitment of protein complexes [29]. Euchromatin structure of embryonic stem cells is a predominant chromatic structure that allows for global gene expression accessibility [176] and facilitates reprogramming to the pluripotent state. It is not surprising that histone modifications might in turn influence the global gene expression by modulating chromatin configuration [177]. Covalent modification of the core histone has a critical role in the regulation of gene expression through acetylation and methylation. Chromatin modification and their function are important especially for gene regulation. Kouzarides (2007) reviewed a number of chromatin modifications characterized by mass spectrometry (for nucleosomal modification) and specific antibodies (for global histone modification) [178]. Cellular condition is the key element for such modifications and these chromatin modifications, as a dynamic procedure, are mediated by a number of histone-modifying enzymes that can fascinate unravels chromatin, recruitment of nonhistone proteins and transcriptional regulation (for review see [178]). Chromatin modifications, to regulate gene expression, are mostly implied be a number of chromatin modifications such as acetylation/deacetylation, phosphorylation, lysine/arginine methylation, deimination, ubiquitylation/deubiquitylation, sumoylatio, ADP ribosylation and proline isomerization which are properly reviewed by Kouzarides (2007) [178].

Histone acetylation is the main type of histone modification during oogenesis, and it is shown that histone acetylation is critical in epigenetic reprogramming [179,180]. For instance, *in vitro* study on acethylation of histones in cloned porcine blastocyst showed that increase the level of acetylation may enhance the embryonic development [181,182]. Generally, hyperacetylation of histone H3 and H4 improve the accessibility of nucleosome to transcriptional machinery [183]. The level of histone acetylation may correlate with the regulation of the expression of genes because more histone acetylation the more expression of a given gene, and vice versa [180]. As mentioned before, histone modifications and DNA methylation are cooperative. Histone modification is able to direct DNA methylation as shown in H3 in Neurospora crassa [184,185] (for review see [186]). Results from recent studies [187,188] have recapitulated that some chromatin modifiers directly act in a *cis*-acting manner [31]. However, a study on the relationship between DNA methylation and histone methylation suggests that they act mostly independently [189]. The affinity of UHRF1 binding protein to the nucleosomal H3K9me3 increases if CpG islands at the nucleosome are methylated but in contrast, in the absence of DNA methylation KDM2A binds to nucleosome having H3J9me3 [190]. Two epigenetic markers, H3K27me3 and CpG DNA methylation, at the *RASGRF1* locus, are interdependent and antagonistic so they are more likely to exclude each other at the same loci [191]. The SNF2 family is an ATP-dependent remodeling complex. In this family, LSH has a role in establishment of normal DNA methylation. A null mutation in Hells gene, codes for LSH that results in the reduction or loss of methylation. Besides, this study suggests the importance of LSH in *de novo* methylation during embryogenesis [192]. Although histone methylation at H3K4 is able to control methylation at DMR of imprinted genes in an allele-specific manner [193,194], it seems to have preventive influence in terms of *de novo* methylation in mammalian somatic cells and may require low promoter methylation [195,196]. Furthermore, mutation in genes, coding for histone methyltransferase such as EZH2 and G9a [197,198] and histone deacetylases like HDAC1 [199] leads to premature death of mammalian embryos typically in less than ten days from fertilization.

## 4. General Features of Imprinted Genes and Their Regulation

After fertilization, a mammalian zygote undergoes proliferation and development. Although there are many active parental genes, involved in a normal embryo development, there are a few genes with bias regulation and transcription, referred to as imprinted genes [61]. Imprinting genes are important for normal embryonic development in mammals. Imprinting genes are selectively (on bias) expressed from a single parental allele [200] and conserved in their molecular structures and epigenomics [75,201]. These genes, essential for normal development, are expressed in a parent-specific manner, regulated by complex epigenetic mechanisms (e.g., DNA methylation, post-translational histone modification) using epigenetic markers (e.g., DNA methylation) [61]. The conflicting interests of parental, imprinted genes are hypothesized as maternally and paternally expressed imprints suppress and enhance the fetal growth respectively (see review [60]). In the nucleus, imprinted genes are mostly placed in a cluster orientation but some are identified as isolated ones [72] such as *Nap1l5*, *Nnat*, *Inpp5f_v2* [202–206] and *Gatm*, *Dcn* and *Htr2a* (for review see [207]). Imprints that are placed within CpG rich region are mostly in clusters, controlled by imprinting the control regions through DNA methylation and histone modifications [58,208,209]. Regulations of imprinted genes are generally proceeded through DNA methylation, post translational histone modification and ncRNAs [210]. In addition, active imprinted genes (expressed allele) contains the allele-discriminating signal (ADS) and the *de novo* methylation signal (DNS) that are necessary for establishing or maintaining methylation [211,212]. For instance, SNRPN is a paternally expressed imprint whose regulation is similar to that of *IGF2r* [212]. Human SNRPB contains two DNS signals; an ADS signal and a signal to maintain paternal imprint (MPI) [213].

### Methylation of Imprinted Genes and Its Abnormalities in Cloned Animals

Short regions of DNA, described as differentially methylated regions (DMRs), are marked by methylation in a parental specific manner and therefore the expressions of such genes are monoallelic. Regulation of clusters of imprinted genes and their activities are mostly controlled by differentially methylated ICRs. In fact, ICRs are DMRs that obtain methylation on one allele (bios) and regulate clustered imprinted genes [138]. In the other words, those DMRs that have a critical role in maintaining imprinting are known as ICRs [81]. CpG spacing suggests a potential influence on ICRs recognition and DMRs methylation in imprints [126]. Moreover, the transcriptional system, especially those traversing ICRs, are considered a common requirement to open chromatin domains, and make targets available for methylation specially in the germ line [32]. Besides, an *in vivo* study in a mouse model illustrated a novel *cis*-acting function for the *H19* ICR [214]. This study shows changes in the size and CpG density that coincide with biallelic expression of the *H19* without any detectable alteration in the methylation pattern. The researchers concluded that, in addition to CTCF sites, there are sequences within the ICR that are essential for its regulatory function. Moreover, the ICR size and CpG density are of determinant elements.

Maternal alleles are dramatically more exposed to ICRs methylation than paternal ones [61,138]. Maternal alleles are mostly methylated on promoters of antisense transcripts but those of paternal alleles are placed between genes (non-promoter regions), suggesting that parental imprinting methylation acts differently [215]. In general, there is higher degree of methylation of the maternal ICRs allele in comparison with that of paternal allele [61]. The *H19* is an example of imprinted genes whose preference is to be expressed from maternal allele. Methylation of the DMDs of *H19*, maternally expressed imprinted gene, is needed for maintenance methylation [141].

DMRs of imprints, mostly, epigenetically signal for monoallelic expression of the gene. *IGF2* encodes a fetal growth-factor and is predominantly expressed from the paternal allele, while *H19* is expressed from the maternal allele and encodes a transcript which may reduce cellular proliferation. In mouse, *IGF2* has a few identified DMRs named DMR0, DMR1, DMR2 and DMR3 among which the first two DMRs are positioned upstream and DMR2 within the *IGF2* gene [216–218]. Recently, an intragenic regulatory DMR has been reported within the last exon of the *IGF2* gene [81]. The comparison between methylation patterns of *IGF2* DMR from parthenogenetic and androgenetic blastocysts on one hand and that evolved from a normal zygote suggests that in normal embryos, paternal allele significantly contributes in the DNA methylation at the locus [81]. Methylation on DMDs of imprints are initially established during gametogenesis and prior to parental pronucleus fusion in the zygote [61,219]. After fertilization, the intergenic DMR of bovine *IGF2* undergoes demethylation followed by low level remethylation before blastocyst stage, which in turn precedes implantation by which the DMR is heavily remethylated. The study speculates that global methylation pattern of SCNT blastocysts is reprogrammed and maintained in a sex specific manner, similar to its normal counterpart. A recent study shows that, except for *RASGRF1* DMR (paternally expressed imprinted gene), methylation of the most imprinted genes during mouse embryonic cleavage stages (preimplantation phase) are mainly controlled by maternal and zygotic DNA methyltransferase 1 (DNMT1) protein family [220].

Abnormalities at imprinted loci have been observed in cloned mammals. In cloned cattle abnormal imprinted gene profiles have been observed especially in the expression of *IGF2* and *H19* [221]. In the *Bos taurus* model, the *IGF2* and *H19* (*IGF2*/*H19*), a conserved cluster of imprinted gene, showed significant variations from the normal pattern, mostly hypomethylation, associated with abnormal expressions of the *H19* (but not *IGF2*) from both alleles in methylation pattern of DMRs [17,222]. Moreover, methylation pattern which mostly occurs in early embryogenesis is dependent on developmental stage and specific to different tissues, as was studied in *IGF2*/*H19* [218].

Super ovulation, also, can cause abnormal imprinting patterns in oocytes [223] that might be attributed to the reduced expression of imprinted parental alleles, *SNRPN*, *PEG3* and *KCNQ1OT1*, but to increased methylation of *H19* [224]. MII oocytes of cloned porcine showed mostly unmethylated profiles of DMR [225]. A recent study on bovine SCNT showed that significant demethylation at the *H19* DMD is attributed to biallelic expression of the imprint which might lead to decline in the rate of implantation [226]. Moreover, biallelic expression of *H19* in bovine is correlated to hypermethylation of the paternal *H19* differentially methylated domain and locus anomalies cause low SCNT efficiency in cattle [226].

## 5. Control of Gene Expression During Gametogenesis

**Figure 2:**
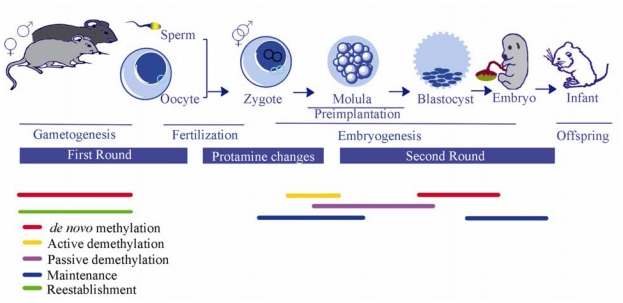
Establishment and maintenance of imprinted genes (epigenetic regulation) during mammalian gametogenesis and development. Sex specific establishment of DNA methylation of imprinted genes occurs during gametogenesis. Just after fertilization, protamine changes occur and follow by the second round of reprogramming begins with embryonic preimplantation. After fertilization, active and passive demethylations happen in parental specific manner. *de novo* methylation happens significantly during both rounds (for review see [61]).

Gametogenesis and embryogenesis involve epigenetic reprogramming to establish proper epigenetic marks and gene regulation. Generally, epigenetic pattern of the genome first reprograms and reestablishes during gametogenesis. The second round of reprogramming and maintenance happens after fertilization, especially during preimplantation of the embryo [61] (Figure 2). Gametogenesis in both sexes involves methylation of DMRs, reestablished in a parent-specific manner [60]. Epigenetic reprogramming is mostly characterized during gametogenesis and early embryonic development especially prior to the zygotic implantation [227]. In fact, during gametogenesis (spermatogenesis and oogenesis) the methylation patterns of these genes are erased and reestablished. These modifications are continued after fertilization and during preimplantation specifically within non-imprinted genes [225] (for review see [180]). Gametogenesis involves sex-specific, epigenetic remodeling of male and female germ lines that matures the gametes for fertilization and constitutes proper regulatory processes [180]. Epigenetic reprogramming in sperm begins with DNA demethylation, followed by DNA remethylation and *de novo* methylation to chromatin modification and histone-to-promatine transition [180,228]. Moreover, during spermatogenesis, testis specific linker histones occupy somatic linker variants. Among the histone variants centromere protein A appears to be epigenetically important during spermatogenesis [180]. Spermatozoa have a transcriptionally inactive and highly condensed chromatin structure. During spermatogenesis in rats, paternal-specific imprinted genes are prone to hypomethylation due to estrogen-associated signaling [229]. In the male germ cells, DMR of *IGF2*/*H19* acquires DNA methylation during spermatogenesis, however, in the female germ cells, the DMR possesses the zinc finger protein CTCF by which the DMR defends against methylation so the allele is able to be expressed [230]. Through fertilization, paternal genome undergoes a series of remodeling events which are controlled by the activity of the oocyte, and the protamine replaced by oocyte-supplied histone and possessing maternal chromatin related proteins [231].

DNA methylation is a sex bias phenomenon. As opposed to the male mouse embryonic germ cells, the female is not that much prone to methylate *IGF2 receptor*, *IDF2* and *H19* [82,232–234]. The same result has been illustrated during the blastocyst stage. It has been shown that in bovine, there is a significant tendency for methylation in the male in comparison to that of the female [81]. Piwi proteins (mili and miwi2) are expressed only in germ line, which are responsible for the establishment of *de novo* DNA methylation in transposons, and it is shown that PiRNAs directs DNA methylation in the male mouse germ cells through which the transposon is silenced [235–237]. In the other words, Piwi/PiRNA complex appears to guide the *de novo* methylation at transposons [235,237] and deactivate transposons within a germline [238]. PiRNAs and siRNAs such as the one located within *AU76*, a pseudogene of *RANGAP1*, negatively regulates transposons through their *cis*-acting function in mouse oocytes as well as establishes the methylation of retrotransposons in the male mouse germ line [235,237,239].

Somatic environment of the male/female germ line shows their influence in DNA methylation of imprints [82]. Using sex-reversed mice to evaluate sex-specific methylation pattern *in vivo*, the germ cells are found to be responsible for female/male imprints during oogenesis/spermatogenesis, though sex chromosome constitution shows significant influence on male germ line for imprint methylation [82]. It seems probable that somatic environment of the genital ridge and that of chromosomal constitution have key roles in the establishment of imprinted genes. *RASGRF1*, paternally expressed imprinted gene, is essential in the male germ line [240,241] suggesting a regulatory mechanism containing DMD methylation and the repeat sequences, by which methylation of the germ line is established [79].

## 6. Epigenetic Regulation During Gestation

Normal fetal development is dependent on proper development of embryo and placenta. These developments are modulated through epigenetic signals during gestation. Although these molecular signals are controlled by the same epigenetic mechanisms, their regulation is independent of each other and may follow different patterns during embryogenesis in comparison to placental development. During pregnancy, most monoallelic expressed genes carry out in extraembryonic tissues, such as trophoblast and yolk sac, regulate the development and function of placenta [44,201]. In placenta, this regulation seems to be directed by histone modification and ncRNAs through DNA methylation [201]. Embryo-placental development is a complex modulating phenomenon through which imprints undergo necessary maintenance, establishment and/or reestablishment. Placental development is under the control of *IGF2* and its degradation receptor, *IGF2r* [242]. *IGF2*, paternally expressed imprint, codes for embryo-placental growth factors. However, its receptor seems an unorthodox, imprinted gene [243].

Embryogenesis involves global methylation to erase and remethylate the methylation pattern. During early embryogenesis, methylation re-establishment occurs mostly within CpG islands and the imprints regulate in a sex-specific manner based on the new gender [79,244,245]. The demethylation mostly happens during primordial germ cells (PGCs) migration towards the genital ridge [79,246,247]. It is hypothesized that histone replacement and chromatin changes, using DNA repair mechanisms, are in accordance with the epigenetic reprogramming of PGC [61]. Evidences for such associations come from the chromatin modification markers, for instance *H3K9me2/3*, *H3K27me3*, *H3K4me2/3*, *H3K9ac*, *NAP-1* and *HIRA*, during early embryogenesis [248].

Just after fertilization, pre-implantation phase, promatines replace with histones and some level of histone modifications occur. Active demethylation of paternal pronucleus of the zygote starts and follows with passive demethylation during the cleavage states. Re-activation of the inactive X chromosome inactivation is the last significant change of the female embryo during pre-implantation development (for review see [43]). Chromatin modifications during germ line development begin with demethylation of imprinted genes in primordial germ cells, in a sex-dependent manner [246]. Then, during female gametogenesis, this modification proceeds to form primary oocytes, and follows to reestablishment of maternal-specific methylation pattern during growth and maturation of oocyte [123]. In male gametogenesis, a number of modification factors are involved. During spermatogenesis, histones undergo hypoacetylation. Especially, DNMTs are significantly important in the alteration of Leptotene to Pachytene. In this transformation, DNA methylation, histone methylation and histone deacetylation are counterparts [249]. The last modification to produce a mature sperm is the promatine formation (for review see [53]).

## 7. Epigenetic Regulation During Embryogenesis

Early embryonic mouse shows high level of DNA methylation and expression of imprinted genes. The epigenetic pattern is maintained in somatic cells but erased in the PGC about 11.5–12.5 embryonic day [61]. At this time, the expression of the imprinted genes in PGC is biallelic and reestablishes during prenatal (in a male embryo) and postnatal stages (in a female embryo) [61,246,250]. Mammalian promoters enriched in H3 K27 trimethylation [251] and H3 K4 trimethylation [252] are mostly occupied by polycomb group (PcG). Besides, PcG proteins influence the pluripotency of embryonic stem cell [253–257]. Study on mouse embryos revealed the regulatory mechanism for imprints in which DNA configuration is the key silencing factor. An imprinted gene, *KCNQ1*, is paternally repressed by ncRNA, *KCNQLOT1*, in association with PcG proteins (EZH2 and Rnf2) at *Cdkn1c*, *Cd81* and *Tssc4 cis* genes [167]. An *in vitro* study on the expression of imprinted genes, *H19* and *SNRPN*, in male mouse [258], suggested a mostly intrinsic, sex-specific reestablishment of DNA methylation (after DNA demethylation) in the male germ line. However, there is still a probability that somatic cells at earlier stages may influence DNA methylation [61].

## 8. Epigenetic Regulation and Placental Development

In mammals, imprinting has an important role in extraembryonic tissue. Their activation pattern seems to be tissue-specific as they are varied between embryonic imprints and placental imprints, for instance in mouse, placental imprints are mostly paternally repressed [201]. The authors suggested an evolutionary relation between placenta imprints and that of chromosome X repression. These findings propose that independent regulatory mechanisms are active in the embryo and in the placenta [259]. The regulatory mechanism suggested for imprints expression, is DNA methylation through histone modification and ncRNAs [201]. Research on a mouse model postulates that the regulation of the expression of imprints is not very firm in the trophoblast as it is in the embryo [260]. In fact, histone methylation maintains the silencing of the inactive allele of the imprints in mouse extraembryonic tissue, placenta. Further, the absence of histone methyltransferase *G9a* that has aberrant effects on placental imprinted cluster, *Kcnq1*. Retrotransposon-derived *Peg 11* (paternally expressed 11) or *Rtl1* (Retrotransposon-like q) is a paternally expressed imprinted gene responsible for the maintenance of placental development especially in fetal capillary development [261,262]. In placenta, repressive histone modification seems more crucial for the maintenance of imprinted genes [201,260]. Moreover, ethanol-induced growth inhibitory effects on the methylation of paternal allele *H19*, suggests CCCTC-binding factor site as a epigenetic switch in placenta [263]. A recent study on methylation status of placental *PEG10* emphasizes the importance of normal methylation of placental imprints for normal development of SCNT in cloned cattle. The placental *PEG10* shows a similar methylation pattern in the cloned calves, which survived and were healthy, in comparison with normal calves. Further, the cloned calves that died because of developmental failure, showed hypermethylation in *PEG10* [264]. The importance of epigenetic regulation for proper placental development is obvious. Thus, unsurprisingly, SCNT paves the way to have a better understanding of placental development and molecular signals that modulate epigenetic regulation.

## 9. Transcriptional Regulation by Polycomb Protein

DNA methylation and PcG proteins are two main silencing epigenetic pathways that are in accordance with each other [265]. PcG proteins are epigenetic regulatory proteins combined in numerous protein complexes as well as individual PcG proteins and usually interact with histones. They are classified as polycomb repressive complexes 1 and 2 classes. EED is a PcG, which can modify histone and change chromatin structure. EZH2, a PcG protein, is a regulatory element for the methylation of CpG in which the protein is in direct contact with DNMTs. PcG proteins play a critical role in epigenetic regulation such as in higher organisms X-chromosome inactivation, imprinting regulation and restoring to pluripotent status [266,267]. Their epigenetic role is more in maintaining chromatin structure as well as reestablishment of transcriptional regulation (for review see [268]), especially during differentiation and development [255,269]. PcG are responsible for maintenance of repression of specific developmental genes [180]. CTCF is an essential factor in insulator’s function that regulates transcription in mammals [270]. It contains 11 zinc-finger DNA binding protein [271] with versatile functions [272,273] as well as a transcriptional activator [274] and a repressor [271,275] (see review [276] for more information). Studies show epigenetic activities of CTCF in regulation of imprints [277] and X chromosome inactivation [148,278,279]. However, post-fertilization methylation of the *H19* ICR in a transgenic mouse model shows the necessity of the CTCF binding sites for the maintenance of the imprint pattern after implantation but not during pre-implantation phase [280]. A recent study on *H19* ICR in SCNT bovine embryos reconfirms that significant demethylation of the gene prevents successful implantation of the embryo [226]. These investigators showed that the CTCF binding sites of the paternal allele are mostly unmethylated, and coincided with the expression of the *H19*, though during postimplantation period the methylation pattern and the expression profile of the gene was similar to control. Transcriptional regulation of genes is also associated with chromatin modification enzymes, such as *HDAC1* [265], *G9a* [281] which are associated with DNMTs [282].

## 10. Conclusion

Creating live and healthy offspring through SCNT technique is only partially explained through epigenetic modifications. Clearly, somatic nuclei need to appropriately reprogram to the pluripotent state from which embryogenesis embarks. Most of the epigenetic modifications are probably mediated by DNA methylation and histone modifications. The epigenetic modifications reviewed here might explain some of the transcriptional regulatory mechanisms in SCNT reprogramming, which could influence embryonic gene expressions and might also affect the placental development during gestation. In contrast to genetic alterations, most epigenetic modifications are reversible, and the modulation of such modifications by reprogramming pluripotent genes in an embryo and placenta increase the amount of successes in animal cloning. In this review, we have highlighted the importance and possible epigenetic modifications that probably influences the efficiency of animal cloning. Proper regulation of these could further influence the life span of the cloned livestock via the epigenetic modulation of somatic gene expression. In addition to unraveling the mechanisms that have been described in the past decade, several other mechanisms would require additional careful investigation. As discussed, somatic profiles of DNA methylation, histone modifications and chromatin configuration must be erased and reprogrammed in a precise manner in terms of both timing and location. Embryo-specific marks must be acquired in cloned embryos, similar to its natural counterpart. For proper acquisition epigenetic marks take place during embryogenesis, and this is why understanding of the epigenetic modification through gametogenesis up to fertilization is crucial. Inappropriate modification and reprogramming would affect the embryo-placental development and, consequently, could lead to failure during gestation, abnormalities and syndromes. This review is intended to emphasize the importance of understanding nuclear reprogramming for proper SCNT and the importance of DNA methylation and chromatin modification in nuclear reprogramming. Failures in the reprogramming will influence normal development of embryo and placenta and cause several abnormalities. All efforts to illuminate the complexity of epigenetic reprogramming that produces healthy cloned offsprings are necessary in order to have a better insight into the interaction between genomics and epigenomics.

## Figures and Tables

**Table 1:** Types of DNA methyltransferases and their epigenetic functions.

DNMT # types [100]	Functions
DNMT1	Maintaining methylation pattern [21,101,102]
	Essential for chromosome replication and repair [21,103,104]
	Essential for *de novo* methylation [105]

DNMT2	Effective in DNA and RNA methylation (for review see [106])

DNMT3a	Establishment of *de novo* methylation pattern [107,108]
	especially during gametogenesis [109]
	Maintaining methylation pattern [101]

DNMT3b	Establishment of *de novo* methylation [107,108]

DNMT3L	Essential for *de novo* methylation [110]
	Enhance *de novo* methylation activity of DNMT3a [111] and DNMT3b [112]
	Establishment of *de novo* methylation pattern especially during gametogenesis [113]

# DNA methyltransferase
